# Marine Fungi Bioactives with Anti-Inflammatory, Antithrombotic and Antioxidant Health-Promoting Properties Against Inflammation-Related Chronic Diseases

**DOI:** 10.3390/md22110520

**Published:** 2024-11-18

**Authors:** Maria-Aliki Papikinou, Konstantinos Pavlidis, Paschalis Cholidis, Dimitrios Kranas, Theodora Adamantidi, Chryssa Anastasiadou, Alexandros Tsoupras

**Affiliations:** 1 Hephaestus Laboratory, School of Chemistry, Faculty of Sciences, Democritus University of Thrace, Kavala University Campus, St. Lucas, 65404 Kavala, Greece; mapapik@chem.duth.gr (M.-A.P.); kopavlm@chem.duth.gr (K.P.); paoholi@chem.duth.gr (P.C.); dikrana@chem.duth.gr (D.K.); theadam@chem.duth.gr (T.A.); 2 Fisheries Research Institute, Nea Peramos, 64007 Kavala, Greece; anastasiadou@inale.gr

**Keywords:** marine fungi, anti-inflammatory, antioxidant, antithrombotic, PUFA, terpenes, alkaloids, lipid bioactives, chronic diseases

## Abstract

Fungi play a fundamental role in the marine environment, being promising producers of bioactive molecules in the pharmacological and industrial fields, which have demonstrated potential health benefits against cardiovascular and other chronic diseases. This review pertains to the analysis of the lipid compositions across various species of marine fungi and their constantly discovered substances, as well as their anti-inflammatory, antioxidant, and antithrombotic effects. The health-promoting aspects of these microorganisms will be explored, through the investigation of several mechanisms of action and interference of their bioactives in biochemical pathways. Despite exceptional results in this field, the potential of marine microorganisms remains largely unexplored due to the limited number of specialists in marine microbiology and mycology, a relatively recent science with significant contributions and potential in biodiversity and biotechnology.

## 1. Introduction

Marine ecosystems are a valuable resource for humans, as they offer numerous direct and indirect benefits to the ecosystem. They play fundamental roles in the control of atmospheric gases, regulation of climate, and biogeochemical cycles of several elements; they provide raw materials and nourishment and are also important for many recreational and cultural activities [1]. The sea is home to a rich biotic community that includes animals, plants, and algae but also many microorganisms like bacteria, protozoa, and fungi. While there is a certain degree of knowledge about the macroscopic world, only during the last decade has a wider focus on the microscopic world for the pharmaceutical and life sciences been achieved. In terms of diversity, seas and oceans represent an almost ‘inexhaustible’ source of microorganisms still unknown and, among these, fungi have received relatively less attention until recently [2].

The first studies referring to marine fungi date back to the mid-19th century, when two new species—*Phaeosphaeria typharum* and *Halotthia posidoniae*—were discovered and isolated from the submerged portions of *Typha* sp. and from the rhizomes of *Posidonia oceanica* [3,4]. In the following years, marine mycological research did not exhibit progress, which explains the research on marine fungi for about 150 years, except for a few sporadic reports. This was due to technical difficulties linked to the collection of samples and the widespread belief that aquatic fungi constituted a marginal aspect, and thus, their presence was occasional and associated with terrestrial contamination phenomena. Only in the second half of the 20th century did some researchers begin to study these organisms systematically and based on the first promising results, a progressively growing number of mycologists focused on sea substrates, leading to the birth of “marine mycology”.

Although in recent years it has been realized that fungi are an essential component of marine ecosystems, having fundamental roles in many processes, ranging from the maintenance of water fertility to the control and regulation of phytoplankton communities. Understanding the biodiversity and ecology of marine fungi, particularly in relation to other living organisms in their ecosystem, is still being studied to assess their benefits to human health and well-being. In addition, these fungi are considered important subjects of research due to their biotechnological potential and practical applications.

Estimates of the global number of fungal species currently present on our planet are quite variable and indicate a range from 2 to over 11 million species. To date, there have been 156,286 species formally described, and among these, only a percentage equal to 1.2% are associated with marine environments (1947 species) [5,6]. Based on the estimates recorded and the information available, it is quite evident that knowledge relating to fungal biodiversity, and especially the marine one, is still quite scarce. In support of this statement, it must be taken into account that new taxonomic entities are attributed practically every time research is undertaken and completed in new marine habitats [7,8].

Based on what has previously been reported and the fact that new species and molecules are continually discovered, research in this area appears extremely promising and could lead to the discovery of several useful compounds, potentially providing solutions to diseases caused by emerging pathogens or those resistant to various synthetic drugs. In the cardiovascular (CV) field related to diseases, occurrences, events, and conditions, there is a series of resources that have identified elements and compounds, which are thoroughly tested as potential treatment mechanisms for CV conditions and diseases caused by chronic inflammation [9].

The primary and secondary metabolites that have been identified from marine fungi include peptides, polyketides, alkaloids, and terpenoids. Many of these identified marine metabolites are bioactive against bacteria, viruses, fungi, protozoa, and tumor cell lines and are currently being studied from the perspective of new pharmaceutical formulations [10,11]. The first marketed product derived from a marine fungus was cephalosporin C, which was isolated from *Cephalosporium acremonium* (now *Acremonium chrysogenum*). Cephalosporin C, is an important antibiotic belonging to the β-lactam family, which blocks synthesis of the bacterial wall and has broad-spectrum bactericidal action [12]. Many other molecules with antibiotic activity, were also described in subsequent years.

This review aims to depict the lipid composition of different species of marine fungi, as well as the presence of significant bioactives, such as terpenes, polyketides, and phenols. Additionally, extensive reference is made to the health-promoting effects related to cardiovascular diseases from bioactives extracted from marine fungi. Lastly, emphasis is also given to the health-promoting effects of marine fungi extracts associated with other pestering diseases and disorders, such as diabetesand cancer.

## 2. Lipid Composition of Marine Fungi

Marine fungi are unique producers of diverse lipids, mainly including constituents like fatty acids, sterols, and other bioactives. Their lipid compositions vary significantly across species and habitats and often display structural adaptations capable of providing them with the ability to survive under extreme marine conditions (e.g., low temperatures, varying pressure, and high salinity). Commonly, marine fungi lipids are high in polyunsaturated fatty acids (PUFAs), including n-3 and n-6 fatty acids, which promote membrane fluidity and protection against environmental stresses while also being high in sterols and other lipophilic compounds that contribute to cell structure stability and support specific metabolic functions. Cellular functions including hormone synthesis, energy generation, and fat storage, in fact, depend heavily on lipid metabolism [13].

Recently, the importance of marine-fungi-derived lipids has also been highlighted for their association with several chronic diseases, as many of these lipids exhibit antioxidant, antimicrobial, anti-inflammatory, antithrombotic, and even anticancer properties. Globally, chronic diseases are a leading source of morbidity and mortality, and interactions between genetic, epigenetic, and environmental variables are present in many disorders. The utilization of substances like lipids in regulating gene expression and providing the individual with diverse health properties is a growing trend thanks to recent developments [14].

Inflammation-related diseases are one of the most common pathologies nowadays [15]. Inflammation is the body’s natural and vital reaction to signals from pathogenic infections or tissue injury. It is crucial for both reestablishing homeostasis and responding to danger signaling deriving from tissue damage. Cardiovascular disorders, autoimmune diseases, and cancer are among the numerous illnesses that have dysregulated inflammatory responses [15,16]. Apoptosis and phagocytosis, for instance, are important processes in resolution events actively carried out by lipids. Therefore, a significant reorganization of lipid metabolic pathways and energy programs occurs throughout the development of pro- and anti-inflammatory processes, which play a key role in the various phagocytic cell polarization states.

Moreover, several lipid bioactives found in marine fungi have excellent responses against specific thrombo-inflammatory signaling and, thus, against several chronic disorders associated to such manifestations, such as lipid vitamins, like vitamin D and A and their vitaminoids and carotenoids; unsaturated fatty acids (UFAs), like omega-3 polyunsaturated fatty acids; amphiphilic polar lipids, including phospholipids and glycolipids, baring the UFAs within their structures; and alkaloids, terpenes, terpenoids, etc. [17].

Because of their unique profiles and bioactivities, marine fungi and their lipid bioactives are great resources for biotechnological applications. Current research especially focuses on extracting and characterizing these lipids for their potential use in pharmaceutical, nutraceuticals, and cosmeceuticals. However, there remains a need for further exploration of the lipid compositions of lesser-studied marine fungi species in order to better understand their potential contributions to health and industry [18].

As aforementioned, marine fungi contain a variety of bioactive compounds derived from lipids that are beneficial for human health, including terpenes, polyketides, and sterols. Lipids, defined by their hydrophobic characteristics, encompass an extensive array of molecules, each with unique properties and functions necessary for any organism. Their synthesis begins with acetyl-CoA, which is transformed into lipids through several biochemical pathways [19]. In marine fungi, the total lipid content was estimated in the species *Clonostachys rosea* 7.9–31.1% of dry weight (DW). In the following subsections, the fatty-acid-, polar-lipid-, neutral-lipid-, and lipid-derived bioactives’ compositions are thoroughly discussed on the basis of a corresponding literature review.

### 2.1. Fatty Acid Composition of Marine Fungi

Fatty acids, which have a carboxylic acid group, and a long chain of aliphatic hydrocarbons are energy sources and biofunctional components in cell membranes. Their biological action affects the metabolism, functionality, and reactivity of cells and tissues to hormones and other signals [20]. They are separated into the following three categories: polyunsaturated fatty acids (PUFAs), monosaturated fatty acids (MUFAs), and saturated fatty acids (SFAs). Marine fungi are rich in SFAs; in particular, several species have been found to have abundant levels of palmitic acid (C16:0) (12.0–78.12% of total fatty acids) and stearic acid (C18:0) (1.34–35.77% of total fatty acids). More specifically, high values of SFAs were observed in the species *P. puberulum* and *Anamorphic fungi* (89.34–90.39%), while in *V. tenerum* it was found that only SFAs existed.

Compared to SFAs and PUFAs, MUFAs (8.38–19.57%) were the lowest subclass of fatty acids, and oleic acid (18:1) was the most prevalent in this group (7.87–56.73%). Furthermore, PUFAs were reported in one study; however, the compositions of the n-3 fatty acids eicosapentaenoic acid (EPA) and docosahexaenoic acid (DHA), well-known for their functions in CV health, were not retrieved. Thus, PUFAs account for 24.19–55.30% of the total fatty acids in these two marine fungi species that were examined in this research. Additional fatty acids that belong under the PUFA class are α-linoleic acid (18:3 n-6) and linoleic acid (18:2 n-6), which are estimated to be between 0.0 and 9.42% and 17.19 and 57.90%, respectively. An overview of the fatty acids detected in several marine fungi is presented in Table 1.

### 2.2. Polar Lipid Composition of Marine Fungi

As opposed to fatty acids, polar lipids (PLs) are characterized by their amphiphilicity, which originates from the existence of both polar and non-polar groups in the structure of the molecule. Specifically, PLs consist of a polar head (phosphate groups, carbohydrates, choline, etc.) and hydrophobic tails, most commonly fatty acids. They are divided into phospholipids and glycolipids. Phospholipids contain a polar phosphate group, and they can be further divided into glycerophospholipids, which are composed of a glycerol backbone, and sphingolipids, which contain a sphingosine molecule as a backbone. On the other hand, glycolipids are a group of polar lipids characterized by the existence of bonds with carbohydrates, and they are further divided into sphingoglycolipids and glyceroglycolipids (galactolipids and sulfolipids). PLs implement important biological processes in the cell [20], while marine PLs have also shown important anti-inflammatory and antithrombotic potential against CVDs and other associated inflammation-related disorders [17,25].

PLs can be found as the main components in cell membranes due to their amphiphilic character, which provides them with the opportunity to form cell bilayers. PL concentrations were measured to range from 4.0 to 32.0 mg/g of DW in several marine fungal strains. Furthermore, 2.0–6.0% and 6.0–18.0% of total lipids, respectively, were made up of glycolipids and phospholipids. Phosphatidylcholines (PCs), a class of phospholipids that includes choline as a headgroup, was estimated for 78.0% of PLs, while other classes of phospholipids, like phosphatidylethanolamine (PE) and phosphatidylinositol (PI), had lower values (6.0% and 0–1.0%, respectively). This study discovered that the values of betaine dodecyl glycerol-3-phosphorylcholine phospholipids were comparable to PE. The polar lipid contents of several marine fungi are presented in Table 2.

### 2.3. Neutral Lipid Composition of Marine Fungi

Neutral lipids are responsible for the energy storage of the cell, and they consist of triacylglycerols (TAGs), diacylglycerols (DAGs), and wax esters. TAGs are composed of a glycerol molecule connected through an ester bond with three fatty acids. Fatty acid methyl esters (FAMEs), which are produced by trans-esterifying fats with methanol, were reported for 0.1–3.0 mg/g of DW marine fungi, whereas the composition of TAGS was estimated to account for 77.0–91.0% of total lipids (TLs), as shown in Table 2.

### 2.4. Lipid-Derived Bioactives of Marine Fungi

Many studies have shown through identification methods and qualitative analysis the presence of terpenes in marine fungi. Terpenes represent a broad category of natural hydrocarbons that function as secondary metabolites, while terpenoids are their oxygenated derivatives that belong to the lipids class. A terpenoid’s biosynthesis is initiated by acetyl-CoA and mevalonic acid’s interaction and involves the assembly of five-carbon isoprene units (C_5_H_8_), usually arranged in a head-to-tail manner; however, alternative construction methods can also be used, incorporating various levels of ring formations, functional groups, oxidation, and unsaturation. Consequently, there exists a wide array of structural classes, and new skeletal forms are continually being identified [27]. Terpenes, moreover, are capable of creating complex structures through several cyclization, backbone assembly and tailoring processes. Although the biological action of many of these compounds has not yet been fully understood, it is known that they act as precursors for a plethora of bioactives, such as steroids, sterols, retinol (vitamin A), and carotenoids. Thus, terpenes take part in the production of significant metabolites, which display defense-related activities against pathogens [28].

**Figure 1:**
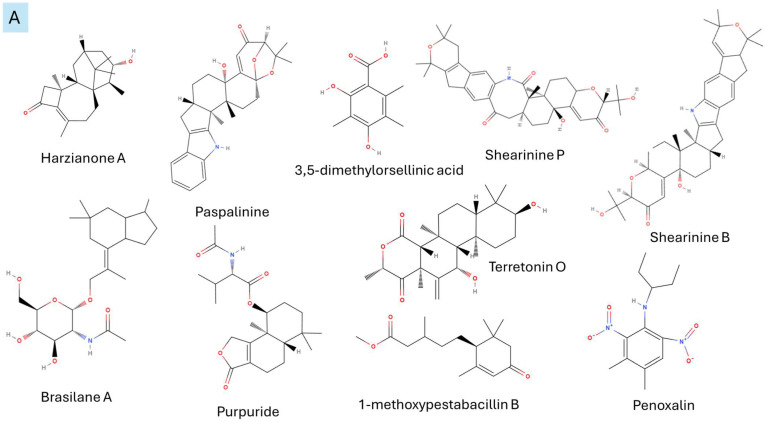

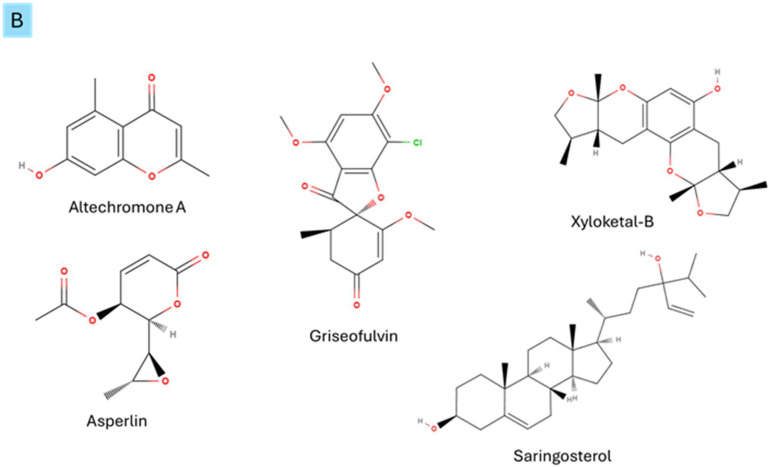
Structures of the most representative terpenoids and polyketides (**A**) and other bioactives with cardioprotective properties (**B**) present in marine fungi (structures were obtained from Molview.org).

It is also important to mention that apart from classic lipid bioactives, like UFAs, MUFAs, PUFAs, PL, lipid vitamins, terpenes, and terpenoids [17], many other bioactives can also be traced in respectable amounts in marine fungi, either more lipophilic ones like sterols or more amphiphilic ones like quinones and phenols. The structures of the most representative bioactives of these classes detected in marine fungi are shown in Figure 1, while the most important terpenes and terpenoids are analyzed in Table 3.

Sterols, as a subgroup of steroids, consist of a tetracyclic cyclopentanoperhydrophenanthrene structure, and their biological function is to maintain and adjust different parameters of the cell membranes, like fluidity, rigidity, and permeability. Marine fungal strains that were dry weighed (DW) contained 10–600 μg/g of sterols, with ergosterol being the most prevalent sterol which, when combined with dehydrostellasterol, comprised 85.0% of sterols. Quinones are composed of hexacarbon cyclic dione with two double bonds. These lipophilic redox compounds act as electron transporters in different biological processes and take part in cell respiration. Lastly, phenols consist of an hydroxyl group (-OH) connected to a carbon atom that is, in turn, part of an aromatic ring. Pigmentation and the reduction in free radicals are some of the features exhibited by phenols.

Apart from the functions of all of these bioactive molecules in the cell, the vast majority display health-promoting effects on human health. For example, PLs have shown antithrombotic activity by inhibiting the biosynthesis of the platelet-activating factor (PAF) [29]. Additionally, phenols are known for their antioxidant activity and their anti-aging and anti-inflammatory action.

**Table 3:** Novel and well-known terpenoids and polyketides extracted from various strains of marine fungus (2019–2024) (structures were loaded from the free databases PubChem, ChemSpider, and Molview).

	Species	Compounds	Structures *	References
Monoterpenes	*Penicillium* sp. SCSIO 41691	1-methyl-12a,12b-epoxyarisugacin M	-	[30]
		1-methyl-4a,12b-epoxyarisugacin M	-	
		2,3-dihydroxy-3,4a-epoxy-12a-dehydroxyisoterreulactone A	-	
		2-hydroxy-12a-dehydroxyisoterreulactone A	-	
		3′-demethoxyterritrems B’	-	
		4a-hydroxyarisugacin P	-	
		1-epi-arisugacin H	-	
	*Diaporthe* sp. SYSU-MS4722	diaporterpene A–C	-	[31]
Sesquiterpenes	*Penicillium* sp. OPR23-FS02	purpuride	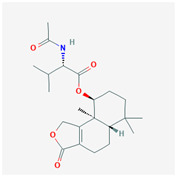	[32]
	*Trichoderma lixii* R22	brasilane A	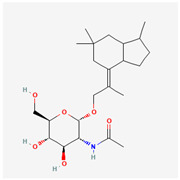	[33]
	*Trichoderma hamatum* Z36-7	5-hydroxyepicyclonerodiol oxide	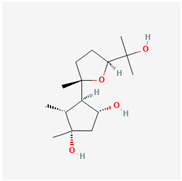	[34]
		4-hydroxyepicyclonerodiol oxide	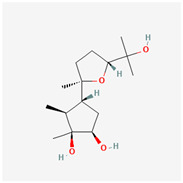	
		trichodermol chlorohydrin	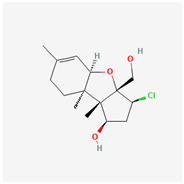	
	*Diaporthe* sp. SCSIO 41011	1-methoxypestabacillin B	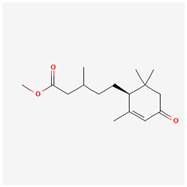	[35]
		11-Nor-8,9*R*-drimanediol	-	
Diterpenes	*Penicillium* sp. OPR23-FS02	penioxalicin	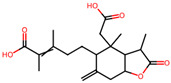	[32]
	*Trichoderma lixii* R22	harzianone A	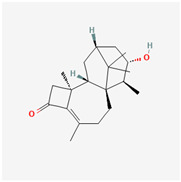	[33]
	*Penicillium* sp. KFD28	7-hydroxypaxilline-13-ene	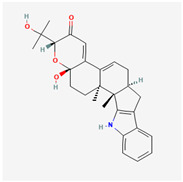	[36]
		paspalinine	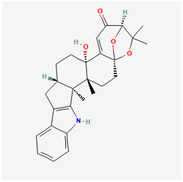	
		paspaline B	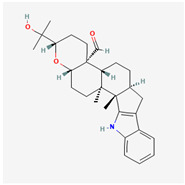	
		pyrapaxilline	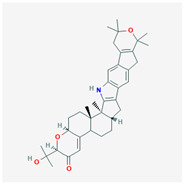	
		shearinine B	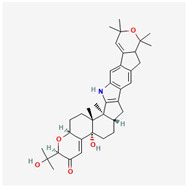	
		shearinine P	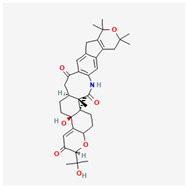	
		3-deoxo-4b-deoxypaxilline	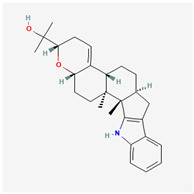	
	*Peroneutypa* sp. M16	solanapyrones	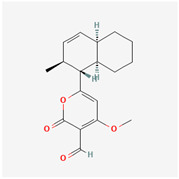 i.e.,	[37]
Meroterpenes	*Aspergillus* sp. CSYZ-1	3,5-dimethylorsellinic acid	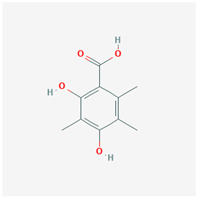	[38]
	*Penicillium* sp. A18	penimeroterpenoids A–C	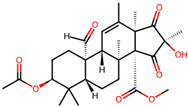 penimeroterpenoid A 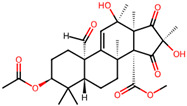 penimeroterpenoid B 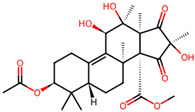 penimeroterpenoid C	[39]
	*Diaporthe* sp. SCSIO 41011	chrodrimanins A/B/E/F/G/H	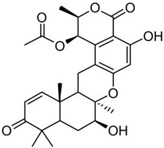 i.e.,	[35]
	*Stemphylium* sp. FJJ006	tricycloalterfurenes E–G	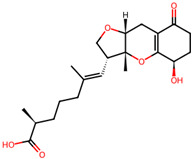 tricycloalterfurene E 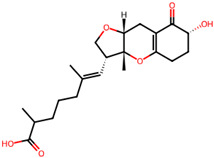 tricycloalterfurene F 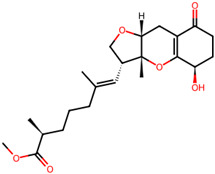 tricycloalterfurene G	[40]
	*Aspergillus terreus* GZU-31-1.	aspermeroterpene A–C	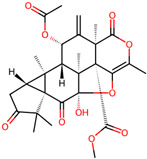 aspermeroterpene A 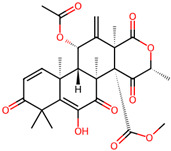 aspermeroterpene B 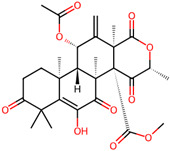 aspermeroterpene B	[41]
		furanasperterpenes A and B	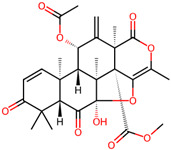 furanasperterpene A 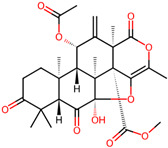 furanasperterpene B	[42]
		11-acetoxy-terretonin E	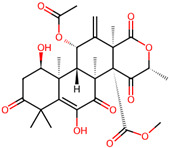	
	*Aspergillus terreus* LGO13	terretonin O	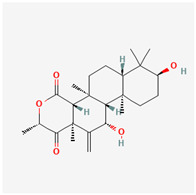	[43]
	*Penicillium bilaiae* MA-267 and *Penicillium chermesinum* EN-480	chermebilaenes A and B	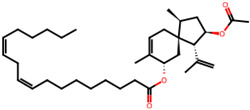 chermebilaene A 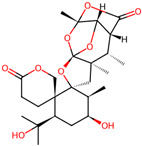 chermebilaene B	[44]
	*Aspergillus* sp. ZYH026	asperaustins A–C	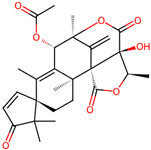 asperaustin A 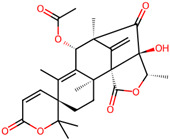 asperaustin B 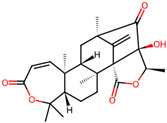 asperaustin C	[45]
Polyketides	*Penicillium* sp. OPR23-FS02	*trans*-3,4-dihydro-3,4,8-trihydroxynaphthalen-1(2*H*)-one	-	[32]
		chloromonilinic acid B	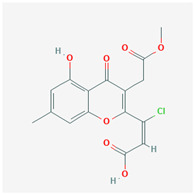	
		chrysophanol	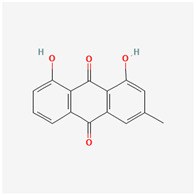	
		emodin	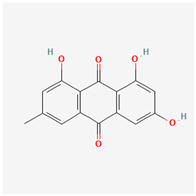	
		ω-hydroxyemodin (citreorosein)	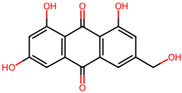	
		questin	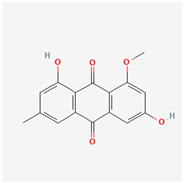	
		endocrocin	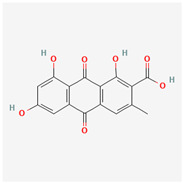	
		ergochrome F	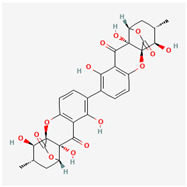	
	*Diaporthe* sp. SYSU-MS4722	acropyrone	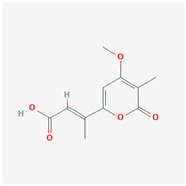	[31]
		nectriapyrone	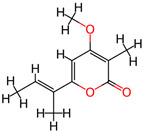	
		monodictyphenone	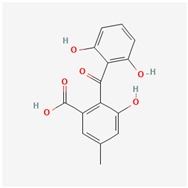	
		2,2′,6′-trihydroxy-4-methyl-6-methoxy-acyl-diphenylmethanone	-	
		pestacin	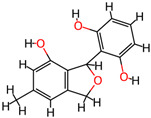	
		3-(2,6-dihydroxyphenyl)-4-hydroxy-6-methyl-isobenzofuran-1(3*H*)-one	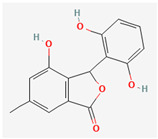	
		3,4-dihydro-6,8-dihydroxy-3-methylisocoumarin	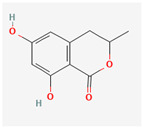	
		2,5-dimethyl-7-hydroxychromone	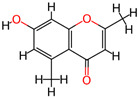	
		4-hydroxyphenethyl-2-(4-hydroxyphenyl) acetate	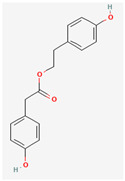	
Anthraquinones	*Aspergillus unguis* 158SC-067 and *A. Flocculosus* 01NT-1.1.5	averantin	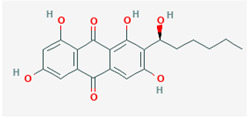	[46]
		7-chloroaverantin	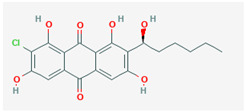	
		1′-*O*-methylaverantin	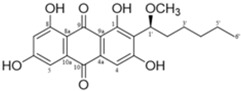	
Bianthraquinones	*Stemphylium* sp. FJJ006	alterporriol Z1–Z3	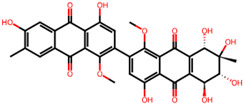 alterporriol Z1 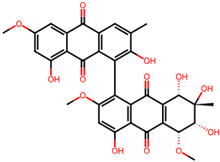 alterporriol Z3	[40]
Phenols	*Aspergillus unguis* 158SC-067/*A. Flocculosus* 01NT-1.1.5	grifolin B	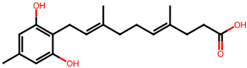	[46]
		12-hydroxyhomovalencic acid	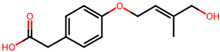	

* Structures were obtained from Molview.org., PubChem, and ChemSpider.

## 3. Marine Fungi Bioactives with Numerous Health-Promoting Properties Against Cardiovascular Diseases

**Figure 2:**
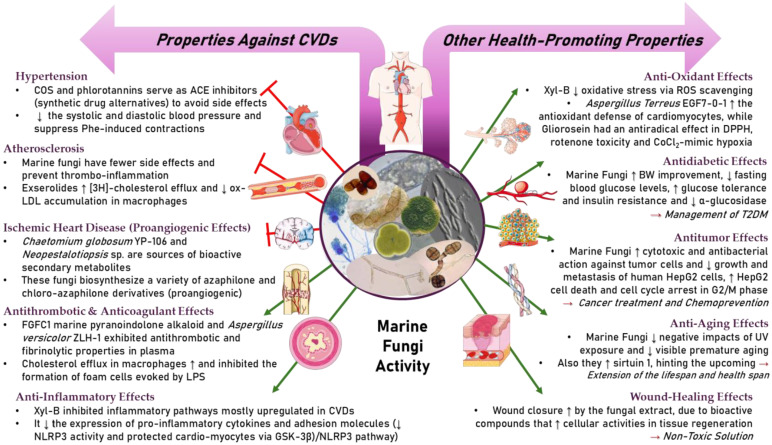
A summary of the cardioprotective benefits and other health-promoting properties of marine fungi and their bioactives.

Cardiovascular diseases (CVDs) are among the most impactful illnesses globally. The available therapeutic options have several side effects, including hypotension, bradycardia, arrhythmia, and alteration in different ion concentrations. Recently, bioactive compounds from natural sources, including plants, microorganisms, and marine organisms, have gained plenty of interest. Marine sources serve as reservoirs for new bioactive metabolites with various pharmacological activities. Marine-derived compounds, especially those from marine fungi, such as n-3 acid ethyl esters, xyloketal B, asperlin, and saringosterol, showed promising results in several CVDs. More specifically, Sajaroketide A, altechromone A, and griseofulvin, three bioactive polyketides isolated from the marine-derived fungal strains KMM4718 and KMM4747 from the sea urchin *Scaphechinus mirabilis*, showed significant cardioprotective effects in an in vitro model of infectious myocarditis, with H9c2 cardiomyocytes co-cultured with Staphylococcus aureus [47]. The structures of the most representative compounds and substances detected in the reported marine fungi are shown in Table 4. Studies, interventions, and clinical trials on the benefits of bioactive extracts derived from marine fungi against cardiovascular diseases are summarized in Table 5, while an overview of the cardioprotective benefits and several other health-promoting properties of marine fungi and their bioactives are depicted in Figure 2.

### 3.1. Marine Fungi Extracts Against Hypertension

Among all cardiovascular diseases (CVDs), hypertension is one of the most serious concerns. It is the leading cause of stroke, ischemic heart disease, dementia, chronic kidney disease, and other CVDs. [48]. Age-standardized prevalence data from 2019 showed that 32% of women and 34% of men aged 30 to 79 had hypertension globally [49]. Numerous natural compounds found in marine species, such as bioactive molecules, chitooligosaccharide derivatives (COS), and phlorotannins, have the potential to serve as angiotensin-converting enzyme (ACE) inhibitors and have been developed into nutraceutical medicinal compounds, which are used to treat hypertension [50,51]. Emphasis is given to natural marine ACE inhibitors as alternatives to synthetic drugs to avoid several serious side effects, as they hold significant potential to become newly emerging therapeutic options for the treatment of hypertension [52]. Xyloketal-B (Xyl-B), a natural pentacyclic fungal marine product, was examined for whether it exerted an antihypertensive effect in a hypertensive rat model, and the corresponding mechanisms were explored. The systolic and diastolic blood pressures in two-kidney, two-clip (2K2C) renovascular hypertensive rats decreased after being supplied with 20 mgkg^−1^d^−1^ Xyl-B for 12 weeks. Moreover, pretreatment with Xyl-B (20 μmol/L) notably suppressed phenylephrine (Phe)-induced contractions in thoracic aortic rings, which were both endothelium-denuded and endothelium-intact, suggesting that both endothelial-dependent and endothelial-independent mechanisms are responsible for the vasorelaxant effect of the drug [53].

### 3.2. Marine Fungi Extracts Against Atherosclerosis

The formation of arterial thrombosis is mostly caused by platelet adhesion under high shear stress, which arises in stenotic atherosclerotic arteries [54]. Atherosclerosis is a chronic, inflammatory, and progressive CVD that is induced by persistent damage to blood vessels resulting from hyperlipidemia and, specifically, elevated low-density lipoprotein (LDL) cholesterol [55]. Compounds derived from marine sources have been beneficial against atherosclerosis. These substances are more effective and have fewer side effects than synthetic ones intending to cure atherosclerosis [56]. PAF is a potent lipid mediator that plays a crucial role in inflammation by activating platelets and repurchasing neutrophils in the process of atherosclerosis, as it operates via the PAF/PAF receptor (PAF-R) pathways. Many medications with marine origins have been studied for their ability to prevent thrombo-inflammation in CVDs. Exserolides increase [3H]-cholesterol efflux and decrease oxidized-LDL (ox-LDL) accumulation in RAW264.7 macrophages via the LXRα-ABCA1 and PPARα pathways, as well as total cholesterol (TC) and triglycerides (TGs) in HepG2 cells and CD36 protein uptake in macrophages [57].

### 3.3. Marine Fungi Extracts Against Ischemic Heart Disease (IHD) and Proangiogenic Effects

Ischemic heart disease (IHD) is caused by insufficient coronary artery blood flow to the heart. The primary mechanism underlying IHD is endothelial dysfunction. Of all CVDs, it is deemed as the primary cause of morbidity and mortality worldwide [58]. In 2019, a report stated that 197 million cases of this disease existed and that it was responsible for 9.14 million deaths worldwide [59]. Marine-derived drugs are better than synthetic ones in treating IHD because of their beneficial action and better results. Three new alkaloids and nine known analogues were extracted from the marine-derived fungus *Penicillium expansum* Y32 and to compounds **4–6**, likely comprising fumiquinazolines or communesins, namely, fumiquinazoline Q (4), cottoquinazoline A (5), and prelapatin B (6), and compounds **8–12**, namely, protuboxepin E (8), protuboxepin A (9), protuboxepin B (10), chaetoglobosin C (11), and penochalasin E (12), were recognized as the principal compounds exhibiting this vasculogenic activity. These compounds could represent a promising therapeutic option for conditions characterized by angiogenesis insufficiency, such as IHD, where the heart muscle’s blood supply is restricted [60]. Studies show that a new deep-sea fungus from the Yap Trench, called *Chaetomium globosum* YP-106 and *Neopestalotiopsis* sp. HN-1-6, has been identified as a possible source of bioactive secondary metabolites with significant implications for CV health applications. These fungi have the ability to biosynthesize a variety of azaphilone and chloro-azaphilone derivatives, which have been demonstrated to possess pro-angiogenic effects in a dose-dependent manner using the zebrafish model [61,62].

### 3.4. Antithrombotic and Anticoagulant Effects of Marine Fungi Extracts

Marine compounds derived from marine fungi may also have antithrombotic and anticoagulant activities. CVDs account for around 20 million deaths worldwide each year, and thrombosis may enhance acute myocardial infarction, IHD, valvular heart disease, peripheral vascular disease, and other CVDs. Studies show that isaridin E concentration-dependently suppressed adenosine diphosphate (ADP)-induced mouse platelet activation, a crucial step in the development of arterial thrombosis and platelet hyperactivity in CVDs associated with an elevated risk of thrombosis. An internal agonist called ADP induced the aggregation, which is essential for physiological hemostasis. While it had no effect on aggregation induced by collagen or thrombin, it was found that isaridin E specifically decreased adenosine triphosphate (ATP) release, platelet activation, and ADP-induced platelet aggregation in a concentration-dependent manner. This finding suggested that isaridin E’s antiplatelet and antithrombotic activities may be attributed to its inhibitory effects on ADP-induced platelet activation and secretion. Safe marine isaridin E, had potent antiplatelet and antithrombotic properties without appreciably altering coagulation parameters, bleeding time, or platelet numbers [63].

The active ingredient fungi fibrinolytic compound **1** (FGFC) 1, a marine pyranoindolone alkaloid small molecule compound, which was extracted from the marine fungal culture *Stachybotrys longispora* FG216, showed antithrombotic, anti-inflammatory, antioxidant, and fibrinolytic properties [64]. The fibrinolytic activity of FGFC1 in plasma, which interfered with the release of fibrinopeptide B to influence protofibril lateral aggregation and raised the vulnerability of clots to fibrinolysis by changing its shape, was demonstrated. It was discovered that FGFC1 can facilitate the FITC-fibrin dissolution in vitro, which is mediated by plasminogen and the single-chain urokinase-type plasminogen activator (scu-PA) [65]. Fibrinolytic enzymes play a crucial role in the management of conditions linked to thrombosis. Aspergillus versicolor ZLH-1, a marine-derived fungus, moreover, yielded versiase, a novel bifunctional fibrinolytic enzyme. Versiase’s anticoagulant activity was evaluated in vitro by timing the clotting of mouse blood. Versiase’s thrombolytic and anticoagulant potencies, as well as its efficient and quick ability to dissolve blood clots, are made possible by its special bifunctional fibrinolytic mechanism. Furthermore, versiase’s low hemolysis rate and minimal effect on human umbilical vein endothelial cell (HUVEC) proliferation led to the conclusion that it is safe for use. Future research will reveal versiase’s thrombolysis and anticoagulant mechanisms, which will open up new avenues in the treatment of thrombosis [66]. Further, from *Aspergillus versicolor* LZD4403, fungus asperlin was isolated, which was then examined for its potential anti-atherosclerotic properties both in vitro and in vivo. Asperlin promoted cholesterol efflux in RAW264.7 macrophages and markedly inhibited the formation of foam cells evoked by lipopolysaccharides (LPS) but not by ox-LDL [67].

### 3.5. Antioxidant Effects of Marine Fungi Extracts

With a range of therapeutic qualities, most notably anticancer and antioxidant activities, marine fungi have become an important and mainly unexplored resource for drug discovery. Recent studies have been conducted, regarding the biological activity of bioactive natural chemicals from fungi against different cancer cell lines and dangerous bacteria. Notably, there are various ways in which a fungal extract with antibacterial and anticancer activities could be useful. *Aspergillus fumigatus* WA7S6, a marine fungus that was found on a sea sponge, seemed to have a variety of potential uses in medicine, in addition to having strong antibacterial and antioxidant properties, and its potential anticancer properties were investigated as well. It was discovered that methyl ester, dehydromevalonic lactone, 9-tetradecynoic acid, and 11-hexadecynoic acid were important compounds with antioxidant action. These results highlight *Aspergillus fumigatus* WA7S6′s diverse therapeutic potential and its applications in the creation of antibacterial, antioxidant, and anticancer drugs. Cancer is a huge global disease burden. Every year, tens of millions of people worldwide are diagnosed with cancer, and more than half of them die as a result of it. The great biodiversity of the marine environment has increasingly piqued the interest of experts, especially in the field of drug discovery. The marine fungus Aspergillus fumigatus WA7S6 has been selected among a group of fungi isolated from marine sponges, as it exhibits a pronounced antimicrobial activity toward a group of pathogenic microbes. The fungus was identified genetically by amplification and analysis of its 18srRNA gene. The fungus’ crude extract was obtained by cultivation of the fungus on rice media. The crude extract was tested for antibacterial activity against a variety of pathogenic microorganisms. The results demonstrated a pronounced antimicrobial action against *P. aeruginosa*, *S. aureus*, *A. niger*, and *Candida albicans*. Furthermore, we tested the antioxidant potential of the Aspergillus fumigatus WA7S6 crude extract using the following three different methods: ATBS, DPPH, and lipid peroxidation assays. The results showed that the crude extract WA7S6 had an IC50 value of 21.35 µg/mL. The anticancer potential of the crude extract was also evaluated against cancer cell lines such as Hela, MCF, and WI-38. The chemical profiling of the fungus extract was identified via GC-mass and in silico molecular docking of the identified compounds on heme oxygenase as a stress protein included in cellular protection, antioxidant, and anti-inflammatory activities, suggesting that some compounds, such as 9-tetradecynoic acid, 11-hexadecynoic acid, methyl ester, and dehydromevalonic lactone, could be relevant for the antioxidant purposes of marine microbes, which can provide fresh approaches to combating oxidative stress and resistant infections, tackling urgent health issues [68,69]. One of the main causes of cardiovascular diseases is an unbalanced state between free radicals and antioxidants in the human body, which results in oxidative stress. One important step in the process of atherosclerotic plaque formation is the oxidative modification of LDL. Therefore, in the case these fungal metabolites possessed antioxidant properties, they would scavenge free radicals and reduce LDL oxidation and, thus, they may be protective against such a sequence of oxidative damage, leading to endothelial dysfunction and the formation of plaques.

According to a recent study, xyloketal B (Xyl-B) is a very potent compound. One of its main mechanisms is reducing oxidative stress, which is a major contributor to the establishment and progression of atherosclerosis and hypertension CVDs, through the scavenging of reactive oxygen species (ROS) and upregulation of the activities of their antioxidant enzymes. This decrease in oxidative stress not only protects the vascular endothelium but also improves overall vascular function [70]. Another study discovered that the *Aspergillus terreus* EGF7-0-1 compound might facilitate an antioxidant defense of cardiomyocytes. By reducing oxidative damage, the compound preserved its structural and functional integrity in myocardial cells, which is vital for preventing future progression of CVDs [71]. Gliorosein, a polyketide separated from a sponge-derived fungus, *Lopadostoma pouzarii*, showed a statistically significant anti-radical effect in the radical scavenging activity test of 2,2-diphenyl-1-picrylhydrazyl (DPPH), but was not particularly potent, due to differing free radical concentrations. Thus, gliorosein apart from DPPH radical-scavenging activity, also exhibited in vitro cardioprotective effects toward rotenone toxicity and CoCl_2_-mimic hypoxia [72].

### 3.6. Anti-Inflammatory Effects of Marine Fungi Extracts

A widespread, nonspecific, and advantageous host response to a foreign challenge or tissue damage is inflammation. Although prolonged inflammation is considered undesirable, it can result in the loss of organ function, including warmth, discomfort, swelling, and redness. In recent decades, a notable increase in interest in marine fungi due to their exceptional anti-inflammatory qualities has been issued. These organisms are found in a variety of marine settings, such as coastal mangroves and deep-sea trenches. They produce a broad range of bioactive chemicals with great promise in the treatment of inflammatory diseases. Numerous new secondary metabolites from fungus living in or on algae, sediments, water, and corals have been reported to possess strong anti-inflammatory properties. Marine fungal compounds have drawn increasing attention due to their distinct method of action, making them of prime interest for the creation of anti-inflammatory medications. Various compounds are isolated from marine fungi, such as alkaloids, terpenoids, polyketides, and peptides. A large proportion of such secondary metabolites, is produced by the species of *Aspergillus* and *Penicillium* [73].

Xyl-B was observed to inhibit some of the main inflammatory pathways, which are mostly upregulated in CVDs. Xyl-B downregulated the expression of pro-inflammatory cytokines and adhesion molecules involved in endothelial dysfunction and atherogenesis. This agent, by suppressing these inflammatory responses, exhibited a preservative action on endothelial integrity and prevented the formation of atherosclerotic plaque [70]. The results for *Aspergillus terreus* EGF7-0-1 sheds light on NLRP3 inflammasome’s activity in the protein complex, related to the inflammatory response. Activation of NLRP3 is linked to the production of inflammatory cytokines and, hence, is damaging to heart tissues. Aspergteroid G was able to downregulate such NLRP3 activity and, thus, formed a base to reduce inflammation and its harmful effects on the heart by protecting cardiomyocytes through the glycogen synthase kinase-3 beta (GSK-3β)/NLRP3 pathway [71].

**Table 4:** Structures of the compounds detected in the referenced marine fungi with a cardioprotective role.

Compounds	Structures *	References
(4) Fumiquinazoline Q	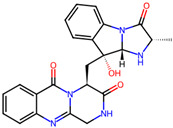	[60]
(5) Cottoquinazoline A	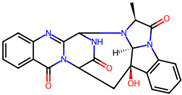	
(6) Prelapatin B	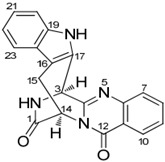	
(8) Protuboxepin E	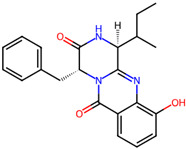	[60]
(9) Protuboxepin A	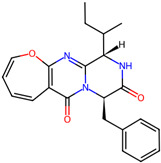	
(10) Protuboxepin B	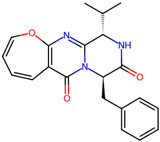	
(11) Chaetoglobosin C	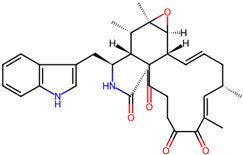	
(12) Penochalasin E	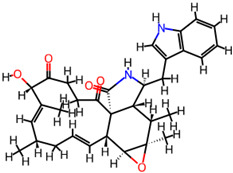	
Isaridin E	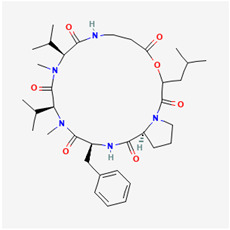	[63]
Methyl ester	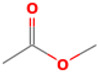	
Dehydromevalonic lactone	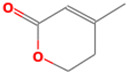	[69]
9-Tetradecynoic acid	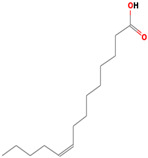	
11-Hexadecynoic acid	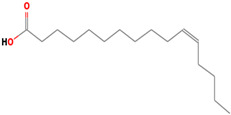	
Asperlin	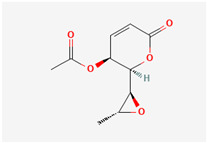	[67]
Aspergteroid G	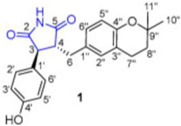	[71]

* Structures were obtained from Molview.org., PubChem, and ChemSpider.

**Table 5:** Studies, interventions, and clinical trials on the benefits of bioactive extracts derived from marine fungi against cardiovascular diseases.

Hypothesis–Intervention	Study Design/Parameters Examined	Main Findings	Structures	Specific Benefits/Other Benefits Observed—Mechanisms of Action(s)	Year	Ref.
*Lopadostoma pouzarii* strain 168CLC-57.3 was isolated from an unidentified marine sponge and cultured for low—molecular weight metabolite isolation	Fungal strain 168CLC-57.3 was isolated from a sponge at Cu Lao Cham, Vietnam. Assays tested DPPH, cell viability, and cardioprotective activities	The isolated compounds, namely, lopouazone A and B, showed weak cytotoxicity against PC-3 cells and H9c2 cardiomyocytes; gliorosein exhibited cardioprotective effects	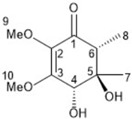 lopouazone A 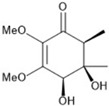 lopouazone B	Gliorosein’s antiradical effect in the DPPH test was statistically significant but not highly potent and may not directly correlate with intracellular ROS action due to differing free radical concentrations	2022	[72]
Isolation of 11 polyketides from the natural complex of the sea-urchin-associated fungi *P. sajarovii* KMM 4718 and *A. protuberus* KMM 4747	The fungal complex, likely from the sea urchin *S. mirabilis* (Sea of Japan), includes co-cultured *P. sajarovii* and *A. protuberus*. Procedures involved DNA extraction, cultivation, urease inhibition assay, and antimicrobial activity tests	Compounds **1** and **2**, namely, sajaroketide A and sajaroketide B were analyzed; some showed cardioprotective effects against *S.-aureus*-induced myocarditis	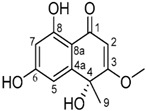 sajaroketide A 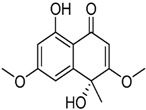 sajaroketide B	Altechromone A increased cell viability by up to 39.9%. Compounds **2**, **6**, and **11** showed no significant effect	2023	[47]
Analyzed XKB and rat Cyp3a2 interactions via homology modeling, molecular docking, and oral XKB’s effects on hepatic Cyp3a	A rat Cyp3a2 homology model was created; XKB’s effects were assessed using high-performance liquid chromatography (HPLC) and luminescence	XKB inhibited Cyp3a-mediated midazolam metabolism in rats, affecting hepatic Cyp3a activity and suggesting possible CYP3A4/Cyp3a2 interactions	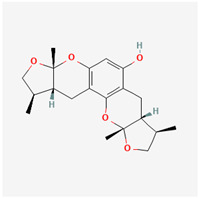 xyloketal B	XKB interacted with the rat Cyp3a2 model via hydrogen bonds. Oral XKB increased midazolam’s area under the curve (AUC) and the maximum achieved concentration of a drug (C_max_) while decreasing oral clearance (CL/F), showing similar inhibition to ketoconazole	2014	[70]
The FGFC1′s effect on fibrin clot structure, lysis, and plasminogen activation using various assays was studied	FGFC1, isolated from *Stachybotrys longispora* FG216 with >98% purity, was extracted from fermentation samples using 2-butanone and ethyl acetate	FGFC1, a marine alkaloid, promoted fibrin dissolution in vitro and effectively treated pulmonary thrombosis in rats	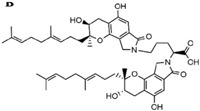 FGFC1	This study showed that FGFC1 had strong fibrinolytic activity in plasma by reducing clot aggregation and increasing lysis sensitivity. It provided a basis for future clinical research on FGFC1 in thrombotic diseases	2023	[65]
Versiase, a fibrinolytic enzyme from *Aspergillus versicolor*, showed anticoagulant potential	*Aspergillus versicolor* ZLH-1, isolated from *Callyspongia* sp., was cultured, and its fibrinolytic enzyme was tested for its activity and toxicity	Versiase’s anticoagulant activity increased with concentration, exceeding 185 s at 100 µg/mL. It showed thrombolytic activity in vitro	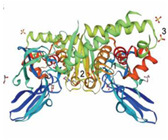 Versiase	Versiase effectively dissolved blood clots with thrombolytic and anticoagulant properties and was safe for use, showing low hemolysis and minimal impact on HUVEC proliferation	2022	[66]
Isaridin E effects on arterial thrombus and the PI3K/Akt pathway were studied in an appropriate mouse model	Mice were treated with isaridin E or clopidogrel before tail transection to measure bleeding time. Hematologic and coagulation parameters were assessed from blood samples after treatment	Isaridin E inhibited ADP-induced platelet activation and aggregation in a concentration-dependent manner, without affecting collagen or thrombin-induced aggregation	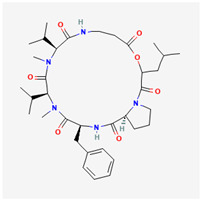 isaridin E	It is safe at 400 μM, with no significant effects on bleeding time or coagulation, making it a promising candidate for treating thrombotic diseases	2022	[63]
This study aimed to explore, *Setosphaeria*-sp.-derived exserolides (I, J, E, and F) to lower lipids by enhancing reverse cholesterol transport (RCT) in vitro	Procedure: Preparation of lipoproteins, cell viability assay, cholesterol efflux assay, determination of intracellular TC and TG levels, and immunoblotting and molecular docking	Exserolide J reduced ox-LDL accumulation and cholesterol efflux via LXRα-ABCA1 and PPARα pathways, decreased CD36 uptake in macrophages, and lowered total cholesterol (TC) and triglyceride contents (TG) in HepG2 cells. Exserolides I, J, and E enhanced PPARα levels; J showed the strongest LXRα interaction, while E had the weakest	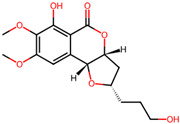 exserolide I 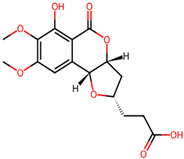 exserolide J 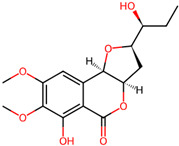 exserolide E 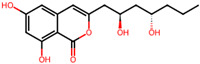 exserolide F	Hyperlipidemia, especially LDL cholesterol, is a key risk factor for atherosclerosis and cardiovascular diseases. Exserolides reduced ox-LDL accumulation and boosted [3H]-cholesterol efflux in RAW264.7 macrophages	2024	[57]
Searching for new drugs to combat CVDs is crucial. Penicillium expansum Y32, isolated from Indian Ocean seawater, may provide lead compounds	Key experiments included extraction, purification, zebrafish heart rate, vasculogenesis, and antiangiogenic vessel growth assays	Compounds **4**, **6**, **8**, and **10**, identified by spectroscopy and quantum electronic circular dichroism (ECD), reduced bradycardia and enhanced vasculogenesis in zebrafish, showing potential for cardiovascular disease	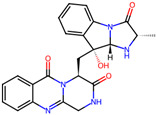 fumiquinazoline Q (4) 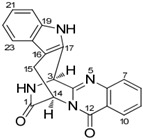 prelapatin B (6) 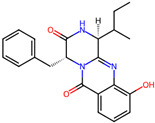 protuboxepin E (8) 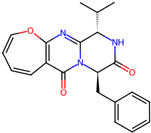 protuboxepin B (10)	Compounds **4**, **6**, **8**, and **10**, namely, fumiquinazoline Q, prelapatin B, protuboxepin E, and protuboxepin B, respectively, promoted vessel growth; others had limited or no activity	2015	[60]
In this search for proangiogenic metabolites, *Chaetomium globosum* YP-106 was isolated from hadal-zone seawater	*C. globosum* YP-106 was isolated from Yap Trench seawater, cultured in 50 flasks at 28 °C, and extracted after fermentation. Spectral data and pro-angiogenic activity were then assessed	Chaetoviridin L (1) and four analogues (2–5) were isolated from *Chaetomium globosum* YP-106 Compounds **1**, **2**, and **5**, namely, chaetoviridin L, chaetomugilin A, and chaephilone D, respectively promoted vascular growth in zebrafish models, while 3 and 4, namely, chaetoviridin E and chaetomugilin O, did not. Compounds **1**, **2**, and **5** are promising for CVD treatment	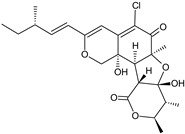 chaetoviridin L (1) 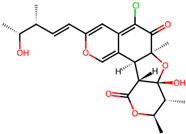 chaetomugilin A (2) 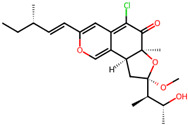 chaephilone D (5) 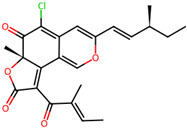 chaetoviridin E (3) 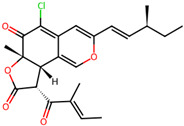 chaetomugilin O (4)	Azaphilones, from deep-sea fungi, may protect against photodamage and may display potential uses as food colorants and UV-proof products	2023	[61]
Four new compounds were isolated from Neopestalotiopsis sp. HN-1-6. Azaphilones (1–3, 5–7) were screened for proangiogenic activity in zebrafish	A fungal strain from Qinzhou was tested for cytotoxicity, antibacterial, and proangiogenic effects using Tg zebrafish embryos	From Neopestalotiopsis sp., three new azaphilones and one phenylpropanoid showed proangiogenic activity and no cytotoxicity	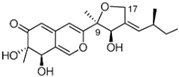 pestaphilone J 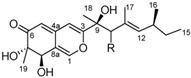 pestaphilone G 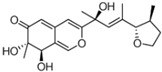 pestaphilone I	Compounds **3**, **5**, and **7**, namely, pestaphilones J, G and I, demonstrated remarkable proangiogenic activities	2024	[62]
Asperlin was isolated from *Aspergillus versicolor* LZD4403. Its anti-inflammatory and atheropreventive activities, were confirmed in high-fat diet (HFD)-fed ApoE−/− mice.	*Aspergillus versicolor* LZD4403 was isolated from a gorgonian and analyzed for anti-inflammatory and lipid effects	Asperlin enhanced cholesterol efflux via PPARγ-ABCA1/G1 and shifted macrophage polarization to M2, indicating potential in atherosclerosis prevention and treatment	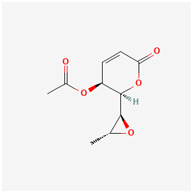 asperlin	Asperlin (1–10 μM) effectively suppressed LPS-induced foam cell formation, comparable to simvastatin, by shifting macrophage polarization from M1 to M2	2017	[67]
Xyl-B, isolated from *Xylaria* sp., was evaluated for hypertension effects using a 2-kidney, 2-clip rat model	Xyl-B, isolated and characterized by HPLC and 1D-nuclear magnetic resonance spectroscopy (D-NMR), was tested in 2K2C rats, with various vascular and cellular assays	This study showed that Xyl-B reduced blood pressure in 2K2C hypertensive rats and induced endothelium-dependent relaxation in aortic rings	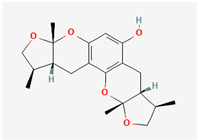 xyloketal B	A limitation of the study is the unverified nitric oxide-soluble guanylate cyclase-cyclic guanosine monophosphate (NO-sGC/cGMP) pathway and Ca^2+^ signaling mechanisms in vascular smooth muscle cells (VSMCs). Caution is needed in interpreting Xyl-B’s antihypertensive effects	2018	[53]
Two new 4,5-disubstituted aspergteroids and four known 4,5-disubstituted analogs were isolated from a soft-coral-associated symbiotic and epiphytic fungus *Aspergillus terreus* EGF7-0-1	Strain EGF7-0-1 was isolated from soft coral in the South China Sea and identified as *Aspergillus terreu* Isolation, quantum chemical calculations, Western blot assays, were conducted	The results suggested that compound **1**, known as aspergteroid G, may reduce the inflammatory response and protect cardiomyocytes through GSK-3β/NLRP3 pathway	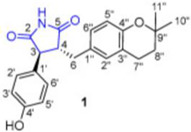 aspergteroid G	The expression of caspase-3 and Bax was regulated, the antiapoptotic gene Bcl-2 was upregulated, the antioxidant capacity of cells was improved, and apoptosis was inhibited	2023	[71]

Structures were obtained from Molview.org., PubChem, and ChemSpider.

## 4. Other Health-Promoting Properties of Marine Fungi Bioactives

**Figure 3:**
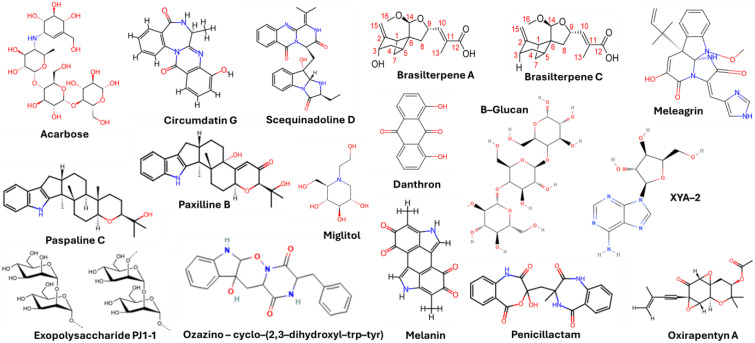
Marine-fungi-derived bioactive compounds and extracts used in the interventions and clinical trials presented in Table 7 and evaluated for their antidiabetic, anticancer, anti-aging, and wound-healing effects.

Marine fungi bioactives have also shown numerous benefits against several inflammation- and oxidative-stress-related manifestations and chronic disorders. The structures of certain compounds contained in marine fungi associated with these diseases are summarized in Table 6 and studies and clinical trials in Table 7. The compounds mentioned in the clinical trials in Table 7, are depicted in Figure 3.

### 4.1. Antidiabetic Effects of Marine Fungi

Recent studies have pointed out that marine-derived fungi exhibit great promise in the development of antidiabetic agents. In this respect, single components isolated from such fungi showed promising in vitro and in vivo activities. Among the most important examples, is exopolysaccharide PJ1-1, an extract of a marine fungus that expressed a potent antidiabetic effect in a Type 2 diabetes mellitus (T2DM) mouse model. The T2DM model was induced in mice with a combination of a high-fat diet and a low dose of streptozotocin, mimicking human diabetes at an early stage. Administration of PJ1-1 resulted in significant improvements in body weight and reductions in the fasting blood glucose levels of the diabetic mice. Over the five-week course of treatment, PJ1-1 decreased the level of fasting blood glucose in a dose-dependent manner; at the highest dose, it decreased by 42.16%. Moreover, PJ1-1 improved glucose tolerance and insulin resistance, which implicated its potential against diabetes [74].

Another marine fungus that has been investigated is *Scedosporium apiospermum* F41-1 for its generation of fumiquinazoline alkaloids, compounds of interest showing insulin-sensitizing activities. These alkaloids have been screened for their ability to induce triglyceride accumulation in 3T3-L1 adipocytes, a cell model often used for studying fat storage and insulin sensitivity. The most important and effective fumiquinazolines, were scequinadolines A, B, D, and E. Among the isolated compounds, the most efficient was scequinadoline D (9) with a half maximal effective concentration (EC_50_) value of 0.27 μM. It was shown that the compound works via the PPARγ pathway, which is one of the most crucial regulators of adipocyte differentiation and insulin sensitivity. Since scequinadoline D activated genes involved in adipogenesis and lipid metabolism, it enhanced sensitivity to insulin and, thus, is a potent candidate for T2DM treatment [75].

The marine-derived fungus *Aspergillus flavipes* HN4-13, isolated from coastal sediments, was found to produce a number of secondary metabolites, including three new butenolide derivatives, namely, flavipesolides A-C, in addition to other known compounds. These metabolites showed considerable antidiabetic activity, especially by the inhibition of α-glucosidase involved in carbohydrate breakdown into glucose. This work revealed that the identified α-glucosidase inhibitors, exhibited potency in different ranges. Compounds **4–6**, namely, 5-[(3,4-dihydro-2,2-dimethyl-2H-1-benzopyran-6-yl)methyl]-3-hydroxy4-(4-hydroxyphenyl)-2(5H) furanone (4), aspernolide (5), and emodin (6), as well as methyl dichloroasterrate (9), acted as non-competitive inhibitors of α-glucosidase, with inhibition constant (Ki) values of 0.43 and 2.8 μM, half maximal inhibitory concentration (IC_50_) values of 19 and 90 μM, respectively. On the other hand, compounds **1–3**, known as flavipesolides A–C, geodin hydrate (8), monomethylosoic acid (10), and epicoccolide B (13) were mixed inhibitors, including both competitive and non-competitive inhibition mechanisms against α-glucosidase. The IC_50_ value of such mixed inhibitors ranged from 9.9 to 95 μM, showing more effective inhibition than well-known antidiabetic drugs like acarbose and 1-deoxynojirimycin, with IC_50_ values of 101 μM and 79 μM, correspondingly. The study pointed out that these compounds, especially butenolide derivatives, demonstrated prominent inhibitory activity against a-glucosidase and low cytotoxicity on the human colon cancer cell line of Caco-2. In view of this, it is an indication that the secondary metabolites from *Aspergillus flavipes* HN4-13 could be a promising leading compound in developing safe and effective antidiabetic drugs [76].

The marine-derived fungus *Paraconiothyrium brasiliense* HDN15-135, isolated from deep-sea sediments, exhibits remarkable antidiabetic potential through its production of unique sesquiterpenoid compounds called brasilterpenes. Out of the five brasilterpenes that were identified, (brasilterpenes A–E), brasilterpenes A and C revealed promising hypoglycemic activities. In an in vivo study with a diabetic zebrafish model, brasilterpenes A (1) and C (3) resulted in a significant decrease in the blood sugar levels of hyperglycemic zebrafish. These compounds exerted their actions by enhancing insulin sensitivity and suppressing gluconeogenesis, a major metabolic pathway by which glucose is synthesized from non-carbohydrate sources. This double action becomes of special relevance in the management of diabetes, since it can help to not only decrease glucose levels in the blood but also to enhance insulin responsiveness of the body, which is reportedly impaired in most diabetic conditions. Furthermore, it was determined that brasilterpene C (3) displayed potent hypoglycemic activity in comparison to the widely used antidiabetic drug rosiglitazone. This result, therefore, suggests brasilterpenes and, especially, brasilterpene C, as leading compounds with strong potential for the development of new antidiabetic therapies [77]. Other deep-sea fungal compounds, such as cladosporol C, tenellone F, ozazino-cyclo-(2,3-dihydroxyl-trp-tyr), penicillactam, and circumdatin G, were identified as inhibitors of α-amylase, α-glucosidase, pancreatic-lipoprotein lipase, hexokinase-II, and protein tyrosine phosphatase-1B, by delaying sugar absorption [78].

### 4.2. Antitumor Effects of Marine Fungi

Marine fungi are a valuable source of natural resources for the creation of anticancer medications due to their unusual structures and distinct functions. Cell cycle arrest is a potentially effective method of halting the growth of cancer cells, since cancer is characterized by an unchecked cell cycle and the uncontrolled proliferation of tumor cells. Therefore, studies have focused on marine compounds that may inhibit human cell proliferation and metastasis and may induce cell apoptosis and cell cycle arrest in the G2/M phase. A major focus of research is the strain *Penicillium* sp. OUCMDZ-1435, known for its contributions to food production, medicinal applications, and natural ecosystems.

Meleagrine, a bioactive substance derived from the fungus *Penicillium* sp. OUCMDZ-1435, has demonstrated strong cytotoxic action against a variety of tumor cells, suggesting that it may have antitumor properties. Meleagrine exhibited antibacterial action, making it a viable option for pharmaceutical research targeted at creating novel antibiotics. This is particularly relevant considering the growing issue of antibiotic resistance. Meleagrin and oxaline have been shown in a study to successfully stop the growth and metastasis of human HepG2 cells, as well as to boost HepG2 cell death and cell cycle arrest in the G2/M phase [79]. Danthron, an hydroxy anthraquinone on the other hand, extracted from the fermentation broth of a marine fungal, is a novel inhibitor of angiogenesis, providing insight into its mode of action. This compound’s antioxidant, antiangiogenic, and anticancer properties pointed to its potential application in cancer treatment and chemoprevention [80]. From the sponge-derived marine fungus *Lopadostoma pouzarii* strain 168CLC-57.3, the new polyketides lopouzanones A and B, as well as the new 1-*O*-acetyl and 2-*O*-acetyl derivatives of dendrodochol B, were isolated. Additionally, the identities of six known polyketides, were determined. All these substances exhibited low cytotoxicity toward PC-3 human prostate cancer cells and H9c2 normal rat cardiomyocytes [72]. A compound derived from a marine fungus, XYA-2, exhibited significant effects on various cellular processes in pancreatic cancer; in addition, it affected the transition of cancer cells during the cell cycle by increasing the apoptotic rate at higher concentrations [81]. Moreover, effective suppression of glioma cell migration, invasion, and proliferation was realized, while apoptosis induction was achieved by oxirapentyn A, which also crosses the blood–brain barrier, according to an in silico analysis [82].

**Table 6:** Structures of certain compounds contained in marine fungi associated with chronic disorders.

Compounds	Structures	References
Exopolysaccharide PJ1-1	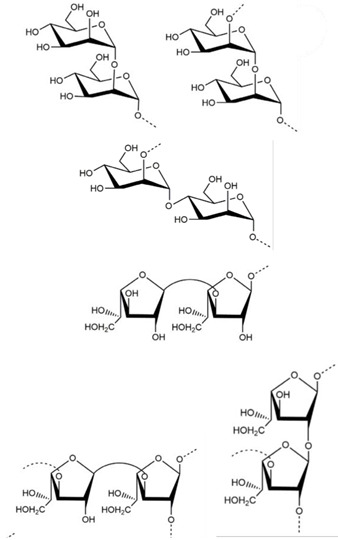 (possible structures of the main repeating disaccharides in PJ1-1)	[74]
Scequinadoline A, B, D, and E (compounds **7–10**—most important and effective fumiquinazolines)	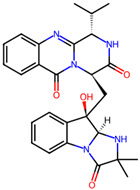 scequinadoline A 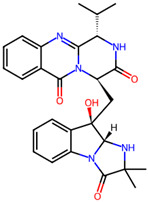 scequinadoline B 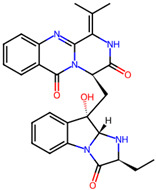 scequinadoline D 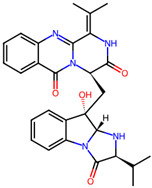 scequinadoline E	[75]
Flavipesolides A–C	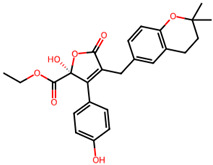 flavipesolide A 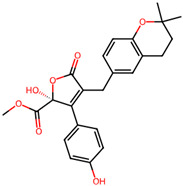 flavipesolide B 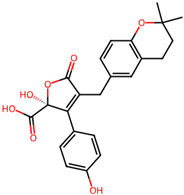 flavipesolide C	[76]
5-[(3,4-Dihydro-2,2-dimethyl-2*H*-1-benzopyran-6-yl) methyl]-3-hydroxy4-(4-hydroxyphenyl)-2(5*H*) furanone (compound **4**) and aspernolide (compound **5**)	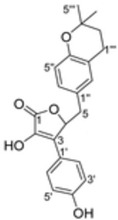 5-[(3,4-dihydro-2,2-dimethyl-2*H*-1-benzopyran-6-yl) methyl]-3-hydroxy4-(4-hydroxyphenyl)-2(5*H*) furanone (4) 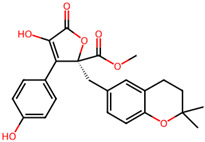 aspernolide A (5)	
Emodin	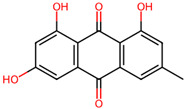	
Geodin hydrate (8), methyl dichloroasterrate (9), and monomethylosoic acid (10)	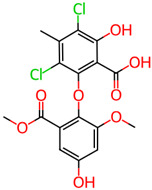 geodin hydrate (8) 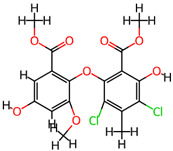 methyl dichloroasterrate (9) 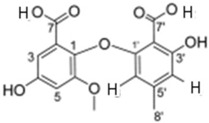 monomethylosoic acid (10)	
Epicoccolide B (13)	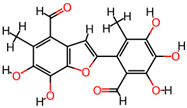	
Brasilterpenes A and C	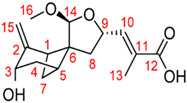 brasilterpene A (1) 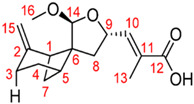 brasilterpene C (3)	[77]
Cladosporol C	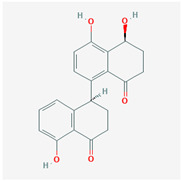	[78]
Tenellone F	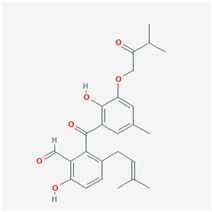	
Ozazino-cyclo-(2,3-dihydroxyl-trp-tyr)	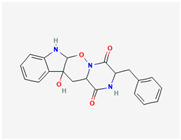	
Penicillactam	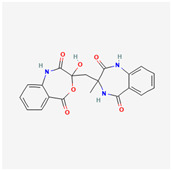	
Circumdatin G	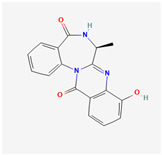	
Meleagrine	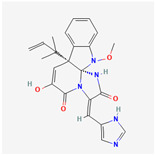	[79]
Oxaline	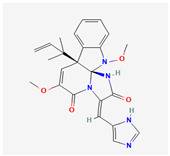	
Danthron	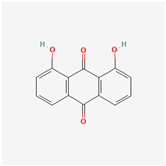	[80]
Lopouzanones A and B	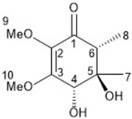 lopouazone A (1) 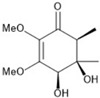 lopouazone B (2)	[72]
1.-.O-acetyl dendrodochol B	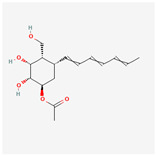	
Oxirapentyn A	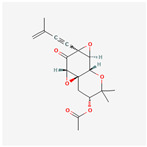	

Structures were obtained from Molview.org., PubChem, and ChemSpider.

### 4.3. Anti-Aging Effects of Marine Fungi

B-glucan is a naturally occurring polysaccharide, found in abundant quantities in the cell walls of several fungi, particularly marine yeast. It belongs to that rare category of compounds with immense anti-aging potential, so it is useful in skincare formulations. B-glucan enhances the intrinsic mechanisms of skin repair, by increasing the retention of moisture and the synthesis of collagen—all vital processes for reducing visible signs of aging like wrinkles, fine lines, and loss of skin firmness. B-glucan extracted from marine yeast was studied with the UV-mutated strain of *Rhodotorula* sp. DAMB1, displaying respectable antioxidant actions. Antioxidants aid in neutralizing free radicals—the main cause of oxidative stress and precocious aging phenomena of skin cells. B-Glucan is, in fact, capable of preserving the youthful appearance of skin by reducing oxidative damage through its action. More importantly, β-glucan has been demonstrated to have excellent skin absorption abilities, which may boost collagen-stimulating activity. Collagen is an important protein that normally promotes skin elasticity and firmness but decreases because of aging. Application of β-glucan topically reestablished collagen levels, hence regenerating firmer and more elastic skin. It also provided protection from UV radiation, one of the comprehensive causes of skin aging, in addition to its anti-aging benefits. Application of β-glucan has been observed to reduce the negative impacts of exposure to UV radiation and to prevent visible premature aging based on environmental factors [83].

In general, this research articulated that *Scopalina hapalia* is a good source of naturally occurring anti-aging agents, since most of its bioactive compounds acted on core key mechanisms involved in the process. More studies are, therefore, recommended, in order to carry on with the isolation and characterization of such bioactive molecules for their potential therapeutic applications [84].

#### Wound-Healing Effects of Marine Fungi

The melanin purified from the halotolerant black yeast *Hortaea werneckii* AS1 was tested for its wound-healing potential using an in vitro scratch assay, a representative model for cell migration and proliferation events occurring during wound closure, against human skin fibroblast (HSF) cells. Observations indicated that the application of melanin did not have a negative impact on tissue regeneration of HSF cells at a dosage of 100 µg/mL. Noticeably, the scratch on HSF cells completely closed at 72 h of incubation regardless of the presence or absence of melanin, indicating that melanin did not hamper the natural process of wound healing of the cells. The known antioxidant and antibacterial activities of melanin should, moreover, account for the potential wound-healing properties of this pigment in cosmetic formulations. The results showed that wound healing was promoted by the fungal extract. This can be attributed to its bioactive compounds that stimulate cellular activities involved in tissue regeneration. The efficacy of the wound healing by the extract is related to its antioxidant and antimicrobial properties, which are projected not only against infection in a wound but also reduce oxidative stress, hence, further improving the healing process. Consequently, this work evidenced that although melanin from *H. werneckii* AS1 did not exert a direct effect on the acceleration of wound closure, it is non-toxic and did not impede the natural process of wound healing, a hypothesis that confirms and supports its candidacy for further exploration for wound-healing applications [85]. These findings open a new avenues in the exploration of this fungus regarding its potential in the development of new therapeutic pathways for better management of wound care.

**Table 7:** Studies, Interventions and Clinical trials of the anti-diabetic, anticancer, anti-aging and wound-healing effects of bioactive extracts derived from marine fungi.

Hypothesis–Intervention	Study Design/Parameters Examined	Main Findings	Specific Benefits/Other Benefits Observed–Mechanisms of Action(s)	Year of Study	References
α-Glucosidase and new inhibitors from *Aspergillus flavipes* HN4-13 are introduced	*Aspergillus flavipes* HN4-13 was isolated from Lianyungang sediment by fermentation and extraction assays	α-Glucosidase inhibitors like acarbose and miglitol treated diabetes but caused side effects	Natural products from *Aspergillus* fungi show anti-tobacco mosaic virus (TMV), DPPH scavenging, α-glucosidase inhibition, and antiviral activities	2016	[76]
This study isolated 3 new and 12 known fumiquinazoline alkaloids from *Scedosporium apiospermum* F41-1 and assessed their antidiabetic potential	*Scedosporium apiospermum* F41-1 was isolated from *Lobophytum crassum* in Hainan, China. Fermentation, extraction, 3T3-L1 differentiation, triglyceride assay, oil red O staining, qPCR, procedures were conducted	The results suggested that scequinadoline D (9) targeted adipocytes and could treat T2DM effectively	Fumiquinazolines and related alkaloids from *Scedosporium apiospermum* F41-1 showed antiviral activity against hepatitis C	2020	[75]
Sixteen indole diterpenoids, including five novel ones, were tested for their cytotoxic and antimicrobial activities	The strain produced a crude extract, which was purified and tested for antimicrobial activity using disk diffusion	Paspaline (C–D)- and paxilline (B–D)-type indole diterpenoids rarely exhibited antimicrobial activity but may have variable cytotoxic effects across different cancer cell lines	Five new and eleven known indole diterpenoids from *Penicillium brefeldianum* WZW-F-69 were observed, highlighting the potential of marine fungi in discovering novel bioactive compounds	2022	[64]
This manuscript presented danthron as a new angiogenesis inhibitor, with antiangiogenic, antitumor, and antioxidant properties	The cell culture included HUVECs treated with danthron or controls. Chick CAM assays and cell cycle analysis followed	Danthron, an hydroxy anthraquinone from a marine fungus, inhibited angiogenesis and tumor cell proliferation by inducing apoptosis	The link between danthron’s antiangiogenic and antioxidant activities warranted further study, supporting its potential as an anticancer drug	2023	[80]
In this study, a homogeneous exopolysaccharide, PJ1-1, was obtained from *Penicillium janthinellum* N29′s fermented broth	C57BL/6 J mice were used; *P. janthinellum* N29 derived from *Acanthus ilicifolius*. Fermentation, composition, and analysis methods, as well as in vivo experiments and fasting insulin assays, were performed	PJ1-1 enhanced glucose metabolism, improved insulin efficiency, and lipid metabolism in T2DM mice, suggesting its potential as an antidiabetic agent	PJ1-1 improved lipid metabolism in T2DM mice by lowering TG, TC, LDL-C, and increasing high-density lipoprotein (HDL)-C	2023	[74]
Five bergamotane sesquiterpenoid derivatives, brasilterpenes, were isolated from *Paraconiothyrium brasiliense* HDN15-135	The fungus was isolated from Indian Ocean sediment. Procedures conducted were fermentation, NMR, glucose assays, toxicity evaluation, and hypoglycemic mechanism studies	Brasilterpenes A (1) and C (3) lowered blood glucose in hyperglycemic zebrafish by enhancing insulin sensitivity	The hypoglycemic activities of compounds **1** and **3** are linked to the S configuration at C-14. Compound **3**, with an hydroxyl group at C-3, is more active	2022	[77]
Β-glucan methyl ester compounds were checked for their applications in the cosmetics field	Assays used for antioxidant, metal-chelating, reducing power, and antibacterial activities.B-glucan was applied in cosmetics and used for almond facial scrub preparation	B-glucan improved skin firmness, collagen production, and has antioxidant, antibacterial effects	Carboxymethylated β-glucan showed greater antibacterial activity against *S. aureus* than the original compound	2024	[83]
Structure-based virtual screening (SBVC) of various deep-sea fungi derived metabolites was performed	Retrieve and prepare protein/ligand structures, virtual screening, molecular docking, molecular dynamics (MD) simulations, residue interaction analysis, and binding free-energy determination, were performed	*Cladosporol C*, *tenellone F*, ozazino-cyclo-(2,3-dihydroxyl-trp-tyr), penicillactam, and circumdatin G were identified as potential inhibitors of key diabetes-related enzymes	Inhibiting α-amylase, α-glucosidase, and hexokinase II (HK-II) delayed sugar absorption, while protein tyrosine phosphatase-1B (PTP-1B) impaired insulin signaling	2023	[78]
Marine fungi are valuable for an antitumor drug development	Annexin FITC/PI assays were used for compound analysis and apoptosis detection L-tryptophan and L-histidine are key precursors in Meleagrin biosynthesis	Challenges in chemical synthesis of bioactive compounds led to the optimization of Meleagrin’s fermentation The initial pH significantly affected the yield; pH 3.0 was optimal for a simpler composition and higher Meleagrin production	These compounds inhibited HepG2 cell proliferation and metastasis, induced apoptosis, and caused cell cycle arrest in G2/M phase	2023	[79]
The study attempted to examine the various valuable medical and environmental utilizations of this beneficial pigment	The purified melanin was evaluated for antioxidant activity, cytotoxicity, in vitro anticancer effects, wound healing, antimicrobial activity, and environmental impact	The pigment showed an antibacterial activity toward *S. aureus* and *A. hydrophila* It can further be applied as an antimicrobial agent against fatal pathogenic strains affecting the aquaculture industry	These findings showed that melanin at a concentration of 100 μg/mL did not influence cell migration	2022	[85]
The impact of XYA-2, a nitrogenated azaphilon previously reported from a deep-sea-derived fungus, on the progression of pancreatic cancer cells was evaluated	Procedures conducted: Cell cycle arrest assay, apoptosis assay, Western blot assay, wound-healing assay, and transwell migration assay	XYA-2 exhibited significant effects on various cellular processes in pancreatic cancer Furthermore, it influenced the transition of cancer cells during the cell cycle, leading to increased apoptosis at higher concentrations	The results from the wound-healing assay, underscored the concentration-dependent inhibitory effects of XYA-2 on the migratory capacity of MIA-PACA2 cells	2024	[81]
In this research on ascidian-derived fungi in the South China Sea, it was discovered that a culture extract of the fungus *Amphichorda felina* SYSU-MS7908 exhibited moderate cytotoxicity against U87-MG human glioma cells	Procedures included: X-ray crystallographic analysis, antiproliferative activity by MTT assay, prediction of blood–brain barrier permeation in silico, cell migration by wound-healing assay, cell invasion by transwell assay, and cell apoptosis assay by flow cytometry	In an anti-glioma assay, oxirapentyn A (7) effectively inhibited the proliferation, migration, and invasion of glioma cells and induced their apoptosis. Furthermore, an in silico analysis suggested that oxirapentyn A has the potential to penetrate the blood–brain barrier	These findings highlight the potential of oxirapentyn A as a candidate for the development of novel antiglioma drugs	2023	[82]

## 5. Materials and Methods

This systematic review was conducted using the Preferred Reporting Items for Systematic Reviews and Meta-Analyses (PRISMA). The Scopus and PubMed databases were used to search for relevant literature. The database search was conducted from April 1, 2024 to August 1, 2024. The keywords used were marine fungi AND lipids, lipid composition, bioactives, polar lipids, neutral lipids, terpenes, fatty acids, PUFAs, n-3 fatty acids, polyketides, alkaloids, peptides, phenols, cardiovascular health, cardioprotective, inflammation, oxidative stress, thrombosis, hypertension, antidiabetic, antitumor, anticancer, antimicrobial, anti-aging, and wound healing.

The articles that were deemed related to the subject were screened according to the following criteria: (i) written in the English language; (ii) research articles; and (iii) published between 2015 and 2024. Firstly, the titles of the articles were considered, followed by the abstract along with the keywords, and then the text thoroughly. From the total number of articles examined, many of them did not meet the criteria and were excluded from this review. The time frame was adjusted further due to the research gap, especially in lipid composition and cardiovascular-promoting effects. For the theoretical part of this review, articles as well as other reviews were examined in a wider time frame of 2005–2024.

## 6. Conclusions

Natural marine products have demonstrated their adequacy against a wide array of diseases, with some possessing novel mechanisms of action and others being the strongest among their inhibitor classes. Innovations in several fields have overcome obstacles related to marine drug discovery and development. Advancements in several procedures, for example, sampling techniques and nanomole structure determination, as well as genome sequencing and mining, upgrade the effectiveness of exploring marine samples for novel therapeutics. Several advanced strategies end up being effective in defeating the supply problem, including total chemical synthesis and microbial fermentation, as well as molecular biology tools. Numerous compounds in different phases of development that effectively use those innovations, highlight natural marine products as the new wave of effective, adaptable, economically, and environmentally friendly drugs.

The marine environment creates a distressing condition, where inhabitants are forced to adapt so as to survive. A large portion of survivors is rich in secondary metabolites, which are restoratively useful. Among the marine organisms, numerous unrefined extracts, improved fractions, and compounds obtained, have exhibited fascinating potential medical advantages over the years. These effects are mediated by compounds from different chemical classes including polysaccharides, terpenoids, phenolic compounds, sterols, carotenoids, alkaloids, and fatty acids.

Owing to a diverse chemical ecology, marine flora are potential sources of bioactive compounds; however, they are the least explored. Regardless of the unprecedented potential for sourcing new prescriptions from natural marine products, very few compounds have actually been utilized for treatment.

Thus, endeavors should be made to develop marine functional compounds responsibly, since their consumption could result in a decrease in the occurrence and gravity of chronic diseases. In order to meet the growing need for a wide range of pharmaceuticals, marine sources hold great promise for providing potent, cheaper, and safer drug candidates toward solving several medical problems and deserve further extensive exploration. This review takes a bird’s-eye view of marine anticancer compounds, both known and yet-to-be discovered, which may be answers to some of our current biological queries and provide novel, safe, and cost-effective solutions in the near future.

## Figures and Tables

**Table 1:** Compositions of fatty acids (%) to the total fatty acids of different marine fungi species.

(% of Total FAs)	16:00	18:00	18:1	18:2	18:3	SFAs	MUFAs	PUFAs	TL% ^1^ (DW)	References
*A. caelatus*	15.12–15.44	6.98–9.12	15.62–25.38	49.47–54.11	2.79–5.29	-	-	-	-	[21]
*E. cladophorae*	15.24–16.70	7.96–19.06	17.76–18.94	40.59–50.17	3.53–4.91	26.06–36.26	18.41–19.57	44.40–55.30	-	[22]
*A. flavus*	13.60–16.34	8.97–12.67	31.04–40.60	29.32–39.20	0.13–1.55	-	-	-	-	[21]
*A. fumigatus*	17.78–19.18	5.07–7.67	19.93–28.35	45.40–54.00	ND ^1^	-	-	-	-	[21]
*P. islandicum*	21.70–40.13	1.34–2.84	12.34–56.73	57.90		23.04–42.97	-	-	-	[23]
*Z. maritima*	24.92–30.36	17.09–35.77	7.87–14.27	17.19–31.83	6.48–9.42	43.62–67.12	8.38–15.18	24.19–40.91	-	[22]
*A. nomius*	18.23–19.87	5.40–6.78	28.34–34.84	35.86–42.84	0–0.90	-	-	-	-	[21]
*A. ochraceus*	19.28–21.12	6.79–8.43	32.06–34.56	36.34–38.32	0–1.25	-	-	-	-	[21]
*A. parasiticus*	15.64–16.44	9.90–15.30	15.14–27.90	40.26–51.52	0.71–4.33	-	-	-	-	[21]
*P. puberulum*	69.18	20.16	10.65	-		89.34	10.65	-	-	[23]
*C. rosea*	12.0–25.0	4.0–12.0	39.0–77.0	-		-	-	-	7.9–31.1	[24]
*V. tenerum*	78.18	21.81	-	-		99.99	-	-	-	[23]
*Anamorphic fungi* (*Agonomycetes*)	33.54–79.12	9.94–11.27	-	-		33.54–90.39	-	-	-	[23]

^1^ ND stands for “not defined”; TL stands for “total lipids”.

**Table 2:** Composition of polar and neutral lipid bioactives from strains of marine fungi.

Polar lipids (mg/g DW)	4.0–32.0
Phospholipids (% of TLs)	6.0–18.0
Glycolipids (% of TLs)	2.0–6.0
PC (% of PLs)	78.0
Betaine-DGTS (% of PLs)	6.0
PE (% of PLs)	6.0
PI (% of PLs)	0–1.0
References	[24,26]
TAGS (% of TLs)	77.0–91.0
FAMEs (mg/g DW)	0.1–3
References	[24,26]
Sterols (μg/g DW)	10–600
Ergosterol (% of sterols)	85.0
Dehydrostellasterol (% of sterols)	-
References	[26]
